# Northern Illinois Dental Society

**Published:** 1897-12

**Authors:** 


### Northern Illinois Dental Society.

To the President and Members of the National Dental Association, and to the President and Members of the National Association of Dental Examiners.

*Gentlemen:*

At the tenth annual meeting of the Northern Illinois Dental Society held at Rockford, Ills., October 20th and 21st, 1897, the undersigned were appointed a committee to draft and present to your Associations suitable resolutions, with a view to remedy an existing evil regarding the interstate practice of dentistry, and we herewith submit the following for your consideration:

Whereas a legal practitioner of any one of the United States, who desires to remove to another State, is, under the existing laws, compelled to comply with certain requirements of the dental law of that State, and

Whereas in many instances such legal practitioner, (sometimes of many years' experience), is subjected to a more or less severe theoretical examination, which can not even be successfully passed by many who are fresh from the college halls, therefore be it

*Resolved*, that the National Association of Dental Examiners and the National Dental Association, be and are hereby requested to enact such rules, or to secure such modification of the dental laws of the various States, which, under reasonable restrictions, will enable competent practitioners to remove from one State to another without being compelled to submit to provisions which are eminently unfair to a large number of capable dentists.

Dated November 10, 1897.

LOUIS OTTOFY,

W. N. TAGGART,

M. L. HANAFORD,

} *Committee.*

Attest: JAMES N. CORMANY, *Secretary.*

